# C-reactive protein levels after 4 types of arthroplasty

**DOI:** 10.3109/17453670903066596

**Published:** 2009-06-01

**Authors:** Hao Shen, Nanxin Zhang, Xianlong Zhang, Weiping Ji

**Affiliations:** ^1^Division of Adult Reconstruction, Department of Orthopedic Surgery, Shanghai Sixth People's Hospital, Shanghai Jiaotong UniversityShanghaiChina; ^2^Department of Orthopedic Surgery, First Affiliated Hospital, Fujian Medical UniversityFuzhouChina

## Abstract

**Background and purpose** Postoperative C-reactive protein (CRP) levels in serum appear to reflect surgical trauma. We examined CRP levels after 4 types of arthroplasty.

**Material and methods** We investigated 102 patients who had total knee arthroplasty (TKA), computer navigation-assisted total knee arthroplasty (NAV-TKA), hip resurfacing arthroplasty (metal on metal, MMSA) and total hip arthroplasty (THA), respectively. CRP levels were estimated before surgery and postoperatively at 2 and 7 days.

**Results** Postoperatively, the peak CRP levels were highest on the second day after surgery in each of the groups. The peak CPR levels after hip resurfacing were lower than those after conventional primary THA. The peak CRP levels after computer navigation-assisted TKA were lower than those after conventional primary TKA.

**Interpretation** The extent of bone and bone marrow injury rather than the region of surgery or the amount of soft tissue damage appears to determine the extent of the postoperative CRP response.

## Introduction

C-reactive protein (CRP), an acute-phase protein, is produced in hepatocytes. CRP is a phylogenetically highly conserved plasma protein that participates in the systemic response to inflammation. Its plasma concentration increases during inflammatory states, a characteristic that has long been employed for clinical purposes (Black et al. 2004). The plasma level of CRP in healthy adults is less than 10 mg/L. The rapid increase in synthesis within hours of tissue injury or infection suggests that it contributes to host defense, and that it is part of the innate immune response (Black et al. 2004). Thus, raised CRP levels are considered to be a useful parameter in detecting complications of bacterial infection after surgery and to reflect the extent of surgical trauma. It has been shown that CRP levels become raised after conventional knee and hip arthroplasty, and that they reflect the degree of systemic trauma after surgery (White et al. 1998, Neumaier et al. 2006). In the recent decades, computer navigation-assisted total knee arthroplasty has been developed for precise alignment; hip resurfacing arthroplasty has been introduced to preserve bone. The severity of systemic trauma from these two types of arthroplasty as compared to that of conventional knee and hip arthroplasty is, however, unknown. The present study was designed to determine the CRP response after these 4 types of arthroplasty.

## Patients and methods

The study was carried out in patients with a diagnosis of osteoarthritis of the hip or knee between January 1 and September 30, 2007. Data were collected prospectively. None of the patients had clinical signs of infection, neoplasia, or had had any operative procedures within 3 months before admission. Analysis of C-reactive protein was done using an immunoturbidometric technique on a Behring BN II autoanalyzer (Dade Behring Inc. Brookfield, CT). Blood specimens were obtained before surgery and on the second and the seventh postoperative days. C-reactive protein levels greater than 10 mg/L before surgery were excluded. The series comprised 115 consecutive patients with 4 types of arthroplasty: hip resurfacing arthroplasty (n = 20), computer navigation-assisted total knee arthroplasty (n = 18), total hip replacement (n = 35), and conventional primary TKA (n = 42). There were 13 postoperative complications, including hematoma (n = 3), deep vein thrombosis (n = 7), secondary wound healing (n = 2), and deep wound infection (n = 1). Patients with any postoperative complications were excluded from the study. This left 102 patients in the study.

The patients in group 1 (n = 19) had hip resurfacing arthroplasty. The metal-on-metal hip resurfacing prosthesis (Conserve Plus; Wright Medical Technology Inc., Arlington, TN) was used for all the patients. Bone cement was used only on the surface of the femoral cup and on the internal surface of the femoral head implant. It was not used around the short stem of the femoral implants. The patients in group 2 (n = 31) had a primary THA. Uncemented metal-on-metal total hip replacement prostheses were used in all patients. The same weight-bearing system (Conserve; Wright Medical Technology) was used in groups 1 and 2, but the femoral stem in group 2 was a tapered system (Profemur; Wright Medical Technology) that was inserted in the femoral cavity. The posterolateral approach was used for both surgeries. Minimally invasive concepts were used routinely for conventional total hip replacement at our institution. In hip resurfacing operations, however, the femoral head was saved and much more soft tissue was released to get better exposure for acetabular preparation (Figure 1) (see Supplementary data). The patients in group 3 (n = 16) underwent computer navigation-assisted total knee arthroplasty. Using the Stryker knee navigation system (Stryker Kneetrac Software version 3.0), the Scorpio condylar total knee prosthesis (Stryker Howmedica Osteonics, Allendale, NJ) was implanted for this procedure without using an intramedullary femoral cutting guide. The patients in group 4 (n = 36) had a conventional primary TKA. An intramedullary femoral cutting guide was used in each case during surgery. The Scorpio condylar total knee prosthesis (Stryker Howmedica Osteonics) was used for all the patients. A parapatellar approach was used both for group 3 and for group 4, but the quadriceps tendon was split much longer in group 3 because the femoral calibration guide was installed in the same approach (Figure 2, see Supplementary data). Patient data are given in Table 1 (see Supplementary data).

### Statistics

Statistical analysis was done using SPSS version 11 for Windows. One-way analysis of variance (ANOVA) was used for comparison between groups at the same time. The level of significance was set at p < 0.05 using a two-tailed test.

## Results

**Figure 3.**
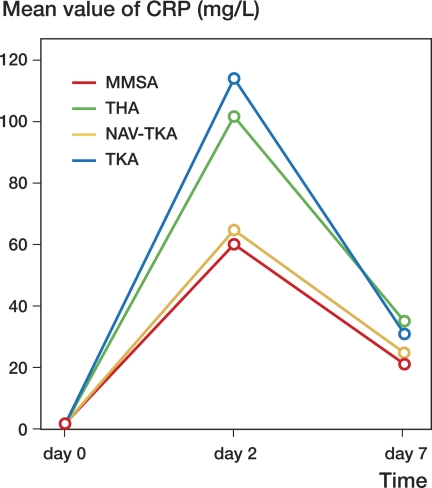
CRP response after 4 different types of arthroplasty.

Before operation, CRP levels were similar in the 4 groups. After surgery, the CRP levels increased in all patients. For each type of operation, the CRP profile after surgery had a characteristic pattern although the maximum amplitude varied between patients. For each group, the peak level of CRP was reached on the second day after surgery, after which it decreased (Figure 3).

**Figure 4.**
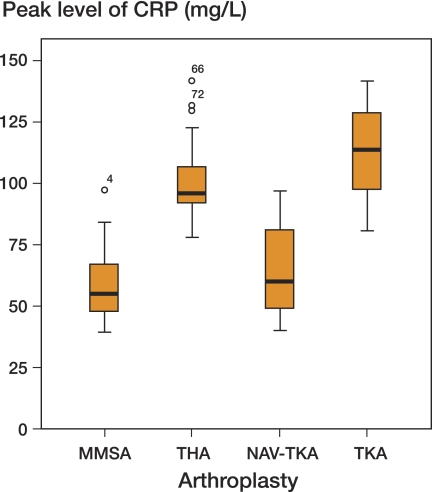
CRP peak levels of 4 types of arthroplasty on the second day. Each box indicates the median, 25, 75 percentiles and minimum and maximum values for each type of arthroplasty.

The peak CRP levels of hip resurfacing patients were statistically significantly lower than those of THA patients. The peak CRP levels of computer navigation-assisted TKA patients were significantly lower than those of TKA patients. We also found significant differences between the peak CPR levels of THA and TKA patients. No statistically significant differences could be seen, however, between the peak CPR levels of hip resurfacing patients and those of computer navigation-assisted TKA patients (Figure 4, Table 2). 

**Table 2.** P-values for the second postoperative day.

	MMSA	THA	NVA-TKA
TKA	p < 0.001	p = 0.02	p < 0.001
NVA-TKA	p = 0.0	p < 0.001	
THA	p < 0.001		

## Discussion

The concentration of CRP in plasma can increase several hundredfold within 24–48 h of tissue injury, from a normal resting state concentration of 5 mg/L (Foglar et al. 1998). A rapid rise in CRP can be observed after orthopedic surgery, reaching the maximum value on the second postoperative day. Then it decreases to the preoperative concentration within about 1 week, irrespective of whether the patient has osteoarthritis or rheumatoid arthritis (Aalto et al. 1984, Larsson et al. 1992, Laiho et al. 2001, Neumaier et al. 2006). If the value remains high or if there is a new rise on days 7 or 8, systemic infection should be suspected (Niskanen et al. 1996). In our study, the shortest hospitalization time of these 4 groups was 7 days. Thus, the postoperative CRP values were determined at the second and the seventh days.

Because no postoperative infections occurred, the increase was interpreted as being solely an effect of tissue damage during surgery. C-reactive protein levels reflect the extent of the surgical trauma, and the C-reactive protein value on the second postoperative day is a crude measure of the degree of operative trauma (Kristiansson et al. 1999, Neumaier et al. 2006). Some authors (Ellitsgaad et al. 1991, Choudhry et al. 1992, Scherer et al. 2001) have reported that C-reactive protein levels depend on the region of trauma or on the extent of surgery, and the CRP levels on the second postoperative day showed the highest power of discrimination between different types of surgical trauma. Another study (Larsson et al. 1992) showed that the increase in CRP depended not only on the amount of tissue injured but also on the type of tissue being damaged—such as bone, fat, or muscle.

Of the 4 different types of arthroplasty in our study, lower peak CRP levels were registered after hip resurfacing and computer navigation-assisted total knee arthroplasty. Compared to the conventional primary THA and TKA, the amount of soft tissue damaged appeared to be much greater during computer navigation-assisted TKA or hip resurfacing, even with the same prosthesis or weight-bearing surface. The reason for this may be that the proportion of medullary cavity tissue damaged is much lower in these two groups. Macrophages, which are important in the development of acute-phase proteins such as CRP, are common in bone/marrow while being less prevalent in muscle tissue (Larsson et al. 1992). In another study (Neumaier et al. 2006), the finding of higher peak CRP levels in patients undergoing arthroplasty than in patients with dynamic hip screws (but not those with proximal femur nails) suggests that trauma to the bone marrow is crucial for the CRP response. Opening of the femoral canal, extrusion of bone marrow from the medullary cavity, and (possibly) loading of the lungs with bone marrow and fat during stem preparation—and often insertion of an endoprosthetic shaft or a femoral nail—similarly results in high CRP levels (Neumaier et al. 2006). Thus, the extent of bone injury and bone marrow injury resulting from different replacements may be crucial in determining the peak levels of the CRP response.

There is no correlation between the C-reactive protein response and age, sex, operation time, amount of bleeding, transfusion, drugs administered, or type of anesthesia (Larsson et al. 1992). However, the region of surgery may be an important factor in determining the peak levels of CRP (White et al. 1998). Peak levels of CRP after TKA have been found to be higher than those after THA (White et al. 1998). The present study has also shown that peak CRP levels after conventional TKA are elevated in comparison with those after conventional THA. There was, however, no obvious difference between peak CRP levels after hip resurfacing and those after computer navigation-assisted TKA. Limited bone and bone marrow injury may be one explanation for the lower peak levels of CRP that were observed after both hip and knee arthroplasty without medullary cavity disturbance in the same region. It is possible that the extent of bone and bone marrow injury during surgery, rather than the location of the trauma, is crucial in determining the extent of the CRP response.

The peak CRP levels after hip resurfacing and after computer navigation-assisted TKA were lower than those after conventional primary THA and TKA; these two types of arthroplasty can be considered “systemic minimally invasive” arthroplasties.
